# Incorporating the thermodynamic effects of temperature and pressure on modeling neuronal gating kinetics

**DOI:** 10.1371/journal.pone.0333592

**Published:** 2025-10-09

**Authors:** Jake A. Miller, Bahram Pahlavan, Bryan Gamboa, Fidel Santamaria

**Affiliations:** 1 Department of Neuroscience, Developmental and Regenerative Biology, College of Sciences, The University of Texas at San Antonio, San Antonio, Texas, United States of America; 2 Air Force Research Laboratory the Human Performance Wing, Human Effectiveness Directorate, Bioeffects Division, Radio Frequency Bioeffects Branch, Joint Base San Antonio Fort Sam Houston, Texas, United States of America; University of South Florida, UNITED STATES OF AMERICA

## Abstract

Temperature and pressure affect neuronal gating kinetics. We recently used thermodynamic macro-molecular rate theory to describe the effects of temperature on the activation rate function of sodium, potassium, and calcium voltage activated conductances. Here, we extend the theory to include the effects of both, temperature and pressure. The theory includes transition changes in heat capacity, entropy, enthalpy, activation volume, expansivity, and compressibility during protein conformation. The complete model replicates experimental results from the literature. We used the expanded model to study how temperature and pressure affect the generation of action potentials in the Hodgkin-Huxley model and in detailed biophysical and morphological models of human cortical neurons. In particular, our results show how pressure can affect the optimal temperature of reaction rates and how small changes in pressure could affect spike timing and correlations across neurons. Our work provides a physics-based approach to adjust reaction rates of neuronal conductances to study cellular function in evolution and under extreme heat and pressure conditions such as those found in blast waves or electro-mechanical neuronal couplings.

## Introduction

Practically all studies on the effects of temperature [1–6] and pressure [7–9] on the activation rate of voltage-gated conductances use an exponential function: either *Q*_10_, the Arrhenius function [10], or its related transition state theory [11]. This approach assumes that temperature only affects the free energy ($\Delta G^{‡}$) of the energy barrier of the activation gating mechanism through constant entropy ($\Delta S^{‡}$) and enthalpy ($\Delta H^{‡}$). However, macroproteins show a universal non-Arrhenius temperature-dependent behavior characterized by a decrease in reaction rate after an optimal temperature (*T*_*opt*_) not due to denaturation [12]. We recently used Macromolecular Rate Theory (MMRT) to demonstrate that sodium (Na), potassium (K), and calcium (Ca) membrane conductances all have *T*_*opt*_ within physiological ranges not associated with denaturation and that the Arrhenius equation produces additive errors in predicting temperature effects [13]. The MMRT assumes that temperature affects both the enthalpy and entropy of the energy barrier through changes in the heat capacity ($\Delta C_{p}^{‡}$) of enzymes.

There is a great deal of interest in understanding how thermodynamic variables affect enzymatic function [14,15]. For example, temperature [16,17], pressure [18–22], and osmotic flow [23,24]. In particular, there are multiple studies to understand how temperature [1,25–28] and pressure [29–32] affect neuronal function. A unified model could be useful for a wide range of applications, such as the effects on cellular function in extremophilic bacteria [33] and deep-sea marine organisms [20,21,34]. This would also be of interest in understanding neuronal function. For example, the heat and pressure waves of a concussive blast [35,36] or electro-mechanical neuronal couplings affecting anesthesia [37,38].

In this study, we extend our MMRT-based approach [13] to integrate the effects of pressure [39], providing a quantitative thermodynamic description of voltage-gated membrane conductances behavior. We first develop the theory and then we evaluate the validity of its parameters through data fitting. We then use the model to understand how the effects of temperature and pressure on voltage gated conductances impact action potential generation, firing frequency, and precise timing. We conclude by exploring physical interpretations of the parameters in the model.

### Theory

#### Macromolecular rate theory.

The value of $\Delta G^{‡}$ between the ground and transition state of an ion channel is:

$$\Delta G^{‡}=\Delta H^{‡}-T\Delta S^{‡}$$ (1)

Changes in heat, $\Delta Q^{‡}$, are related to $\Delta H^{‡}$ and $\Delta S^{‡}$ via $\Delta C_{p}^{‡}$ when $\Delta P^{‡}=0$:

$$d\Delta Q=\Delta C_{p}^{‡}dT=Td\Delta S^{‡}=d\Delta H^{‡}$$ (2)

Integrating assuming constant $\Delta C_{p}^{‡}$ [17], to get $\Delta S^{‡}$ and $\Delta H^{‡}$ and substitute in Eq 1, we get the basis of MMRT.

$$\Delta G_{MMRT}^{‡}=\Delta C_{p}^{‡}(T-T_{o})-\Delta C_{p}^{‡}T\ln\left(T/T_{o}\right)\;-T\Delta S_{o}^{‡}+\Delta H_{o}^{‡}$$ (3)

where $\Delta S_{o}^{‡}$, and $\Delta H_{o}^{‡}$ correspond to a reference temperature *T*_*o*_.

The rate coefficient function is based on the Eyring equation, Eq 4.

$$k=\frac{k_{B}T}{h}e^{-\Delta G^{‡}/RT}$$ (4)

where *k*_*B*_ and *h*, are Boltzmann’s and Planck’s constants, respectively, and *R* is the universal gas constant. The value of *T*_*opt*_ is where $\frac{dk}{dT}=0$ [13,16,40].

#### Incorporating the effects of pressure.

The effect of pressure, *P*, on a reaction rate is mediated by the activation volume, $\Delta V^{‡}$ [41,41–44]:

$$k_{P}=k_{o}e^{-(P-P_{o})\Delta V^{‡}/RT}$$ (5)

Several studies suggest a positive change in $\Delta V^{‡}$ for ion channel opening conformations [29–32,45–48]. Based on [39] and [19,21,22]:

dG=−SdT+VdP (6)

Entropy depends on temperature and pressure

$$dS={\frac{\partial S}{\partial T}|}_{P}dT+{\frac{\partial S}{\partial P}|}_{T}dP$$ (7)

Using Eq 2 for $\Delta C_{p}^{‡}$ and the Maxwell relation ${\frac{\partial S}{\partial P}|}_{T}=-{\frac{\partial V}{\partial T}|}_{P}$ we get:

$$dS=\frac{C_{p}}{T}dT-{\frac{\partial V}{\partial T}|}_{P}dP$$ (8)

The isobaric thermal volume is $\hat{\alpha }=\alpha V$ with *α* the expansivity coefficient:

$$\alpha =\frac{1}{V}{\frac{\partial V}{\partial T}|}_{P}$$ (9)

Thus,

$$dS=\frac{C_{p}}{T}dT-\hat{\alpha }dP$$ (10)

Similarly for volume:

$$dV={\frac{\partial V}{\partial T}|}_{P}dT+{\frac{\partial V}{\partial P}|}_{T}dP$$ (11)

Using the isothermal volume compressibility, $\hat{\kappa }=\kappa V$, with the compressiblity coefficient, $\kappa =-{\frac{1}{V}\frac{\partial V}{\partial P}|}_{T}$, we get

$$dV=\hat{\alpha }dT-\hat{\kappa }dP$$ (12)

Assuming that *C*_*p*_, $\hat{\alpha }$, and $\hat{\kappa }$ are temperature and pressure independent, the integrals are:

$$S-S_{o}=C_{p}\ln\left|T/T_{o}\right|-\hat{\alpha }(P-P_{o})$$ (13a)

$$V-V_{o}=\hat{\alpha }(T-T_{o})-\hat{\kappa }(P-P_{o})$$ (13b)

Where *T*_*o*_, is a reference temperature with associated reference values *P*_*o*_, *S*_*o*_ and $V_{o}$. We substitute in Eq 6.

$$G(T,P)=C_{p}(T-T_{o}-T\ln\left|T/T_{o}\right|)-S_{o}(T-T_{o})\;+\hat{\alpha }(T-T_{o})(P-P_{o})+V_{o}(P-P_{o})\;-\frac{\hat{\kappa }}{2}(P-P_{o}{)}^{2}+G_{o}$$ (14)

We can convert this into values for the change between the ground and the transition state of the reaction. Because MMRT uses $\Delta H_{o}^{‡}$, we can make the substitution $G_{o}=H_{o}\;-\;T_{o}S_{o}$ and ultimately produce:

$$\Delta G^{‡}=\Delta C_{p}^{‡}[(T-T_{o})-T\ln\left|T/T_{o}\right|]-T\Delta S_{o}^{‡}\;+\Delta {\hat{\alpha }}^{‡}(T-T_{o})(P-P_{o})+\Delta V_{o}^{‡}(P-P_{o})\;-\frac{\Delta {\hat{\kappa }}^{‡}}{2}(P-P_{o}{)}^{2}+\Delta H_{o}^{‡}$$ (15)

Where $\Delta {\hat{\alpha }}^{‡}=\Delta {\left(\alpha V\right)}^{‡}$ and $\Delta {\hat{\kappa }}^{‡}=\Delta {\left(\kappa V\right)}^{‡}$. Eq 15 incorporates the terms from Eq 3 and Eq 5. To obtain the kinetics, we plug Eq 15 into Eq 4.

The activation volume as a function of pressure and temperature is found in Eq 13b as:

$$\Delta V^{‡}=\Delta {\hat{\alpha }}^{‡}(T-T_{o})-\Delta {\hat{\kappa }}^{‡}(P-P_{o})+\Delta V_{o}^{‡}$$ (16)

The value of *T*_*opt*_ is:

$$T_{opt}=[\Delta C_{p}^{‡}T_{o}-\Delta H_{o}^{‡}+\Delta {\hat{\alpha }}^{‡}T_{o}(P-P_{o})\;-\Delta V_{o}^{‡}(P-P_{o})+\frac{\Delta {\hat{\kappa }}^{‡}}{2}(P-P_{o}{)}^{2}]/(\Delta C_{p}^{‡}+R)$$ (17)

## Materials and methods

We used our previous parametrization of MMRT for the values of $\Delta C_{p}^{‡}$ and $\Delta H_{o}^{‡}$ [13]. As we did before, the values of $\Delta S_{o}^{‡}$ were adjusted so *k* = 1 at $T=20^{\circ }$C. The reference temperature and pressure were $T_{o}=25^{\circ }$C and *P*_*o*_ = 1 atm, respectively.

We conducted a literature search for experimental data on the effects of pressure on the kinetics of voltage-gated channels[29–32,45,47–49]. We used the results from these papers to determine the other parameters, see Table 1.

**Table 1:** Parameter values for generating figures based on the thermodynamic model. The values for $\Delta C_{p}^{‡}$, $\Delta S_{o}^{‡}$, and $\Delta H_{o}^{‡}$ were obtained from our previous publication [13].

	Na	K	Ca	Units
$\Delta C_{p}^{‡}$	−2.76±0.92	−1.70±0.59	−5.07±3.58	kJ mol^−1^
$\Delta S_{o}^{‡}$	−113±38.90	−130±7.77	−2.30±138	J mol^−1^ K^−1^
$\Delta H_{o}^{‡}$	33.90±1.40	31.80±2.06	70.80±1.11	kJ mol^−1^
	$5^{\circ }$C	$10^{\circ }$C	$15^{\circ }$C	Units
$\Delta V_{Na}^{‡}$	44	35	30	cm^3^ mol^−1^
$\Delta V_{K}^{‡}$	42	37	31	cm^3^ mol^−1^
		High	Low	Units
$\Delta {\hat{\alpha }}^{‡}$		–1	10^−2^	cm^3^ mol^−1^ K^−1^
$\Delta {\hat{\kappa }}^{‡}$		10^4^	10^2^	cm^3^ mol^−1^ GPa^−1^
				Units
*T*_*o*_		25		°C
*P*_*o*_		1		atm
$\Delta V_{o}^{‡}$		19		cm^3^ mol^−1^

Studies on the squid’s giant axon [30–32] reported values of $\Delta V^{‡}$, Table 1. We assumed a linear relation between reference activation volume and temperature, which resulted in $\Delta V_{o}^{‡}=19{\text{cm}}^{3}{\text{mol}}^{-1}$ at *T*_*o*_. The values for $\Delta V_{Na}^{‡}$ and $\Delta V_{K}^{‡}$ were very close to each other [30,31], so we used their average for the simulations.

From the values of $\Delta V_{Na}^{‡}$ and $\Delta V_{K}^{‡}$ at different temperatures and using Eq 16 we calculated an average value of $\Delta {\hat{\alpha }}^{‡}=-1{\text{cm}}^{3}{\text{mol}}^{-1}{\text{K}}^{-1}$, which is consistent with an earlier report [47]. The change in *α* across an increase in temperature was found to be negative [50,51] but positive for unfolding [51]. So, we might also estimate $\Delta {\hat{\alpha }}^{‡}$ at a lower $10^{-2}{\text{cm}}^{3}{\text{mol}}^{-1}{\text{K}}^{-1}$. Therefore, we estimate a high magnitude value of $\Delta {\hat{\alpha }}^{‡}=-1$ and a low value of 10^−2^.

From $\Delta {\hat{\alpha }}^{‡}=\Delta {\left(\alpha V\right)}^{‡}$ approximate $\Delta {\hat{\alpha }}^{‡}\approx \alpha \Delta V^{‡}\;+\;V\Delta \alpha^{‡}$. Since the scale of $\Delta V^{‡}$ is 10^2^, and assuming both terms are approximately equal or the first dominates, the scale of $\alpha \approx 10^{-2}{\text{K}}^{-1}$. This is near reported values of *α* in the $10^{-4}\;-\;10^{-3}{\text{K}}^{-1}$ range [50–53] and will be fine for analyzing the impact of this parameter.

Dreydroppel et al. [39] provides a value for $\Delta \kappa^{‡}$ of 1.8 GPa^−1^ with $\Delta {\hat{\kappa }}^{‡}$ of $130{\text{cm}}^{3}{\text{mol}}^{-1}{\text{GPa}}^{-1}$. Others reported compressibilities on the scale of $0.1{\text{GPa}}^{-1}$ [50,52,54]. Assuming the same $\Delta V^{‡}$ as in [39] suggests a high value of $\Delta {\hat{\kappa }}^{‡}=10^{4}$.

Rapid changes in pressure could result in increases in temperature, known as adiabatic heating. Based on previous reports [31,46,47,49] we assume an adiabatic heating of $1^{\circ }\text{C}$ per 20MPa (≈200atm).

We tested the thermodynamic effects of pressure and temperature on membrane conductance gating on neuronal models of action potential generation. First, we used a Hodgkin-Huxley system of equations. We multiplied the reaction rate of each conductance by the rate coefficient *k* normalized by modifying $\Delta S_{o}^{‡}$ to the control experimental condition of $6.3^{\circ }\text{C}$, Eq 4. In another set of simulations, we chose four models from the Allen Brain Cell Types database. We used an identical approach as in our previous publication [13]. All simulation files, analysis scripts, and data are available in github.com/SantamariaLab or by request. They are also in ModelDB data base Model Number 2019887.

## Results

### Pressure effects on rate coefficient function

**Fig 1:**
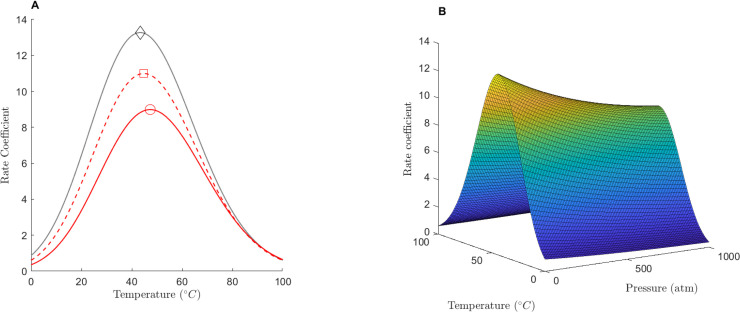
The effects of pressure on the reaction rate coefficient function of average sodium conductances. (**A**) Reaction rate coefficient vs temperature at 1 atm (⋄), average ocean depth ≈350 atm (◻), and Mariana trench ≈1,072 atm (∘). (**B**) The rate coefficient as a function of pressure and temperature. Parameters were: $\Delta C_{p}^{‡}=-2.76\times 10^{3}\text{ kJ}{\text{mol}}^{-1}$, $\Delta S_{o}^{‡}=-113\text{ J}{\text{mol}}^{-1}{\text{K}}^{-1}$, $\Delta H_{o}^{‡}=34\times 10^{3}\text{ kJ}{\text{mol}}^{-1}$, and $\Delta V_{o}^{‡}=19\;{\text{cm}}^{3}{\text{mol}}^{-1}$, $\Delta {\hat{\alpha }}^{‡}=-0.20,1\times 10^{-2}$ and $\Delta {\hat{\kappa }}^{‡}=1\times 10^{2}\;{\text{cm}}^{3}{\text{mol}}^{-1}{\text{GPa}}^{-1}$.

To gain intuition on how pressure affects the reaction rate coefficient of membrane conductances we plotted *k* (Eq 4 with Eq 15) as a function of temperature and pressure. We used our previously calculated averaged temperature thermodynamic parameters for Na conductances ($\Delta C_{p}^{‡}$, $\Delta S_{o}^{‡}$, $\Delta H_{o}^{‡}$) and our estimated value of $\Delta V_{o}^{‡}=19\;{\text{cm}}^{3}{\text{mol}}^{-1}$. For the expansivity and compressibility we used $\Delta {\hat{\alpha }}^{‡}=-0.20\;{\text{cm}}^{3}{\text{mol}}^{-1}{\text{K}}^{-1}$, and $\Delta {\hat{\kappa }}^{‡}=1\times 10^{2}\;{\text{cm}}^{3}{\text{mol}}^{-1}{\text{GPa}}^{-1}$ because this combination resulted in decreasing values of the reaction rate as a function of pressure, consistent with experimental reports, see Table 1 and Methods. We plotted the value of *k* at three representative pressures (atmospheric pressure, 1atm; average ocean depth, ≈370atm; and bottom of the Mariana Trench, ≈1,072atm), Fig 1A. With this combination of parameters the value of *T*_*opt*_ varied over a small range, from $T_{opt}=38.25^{\circ }\text{C}$ at 1 atm to $T_{opt}=40.96^{\circ }\text{C}$ at 1,000 atm, Fig 1B.

**Fig 2:**
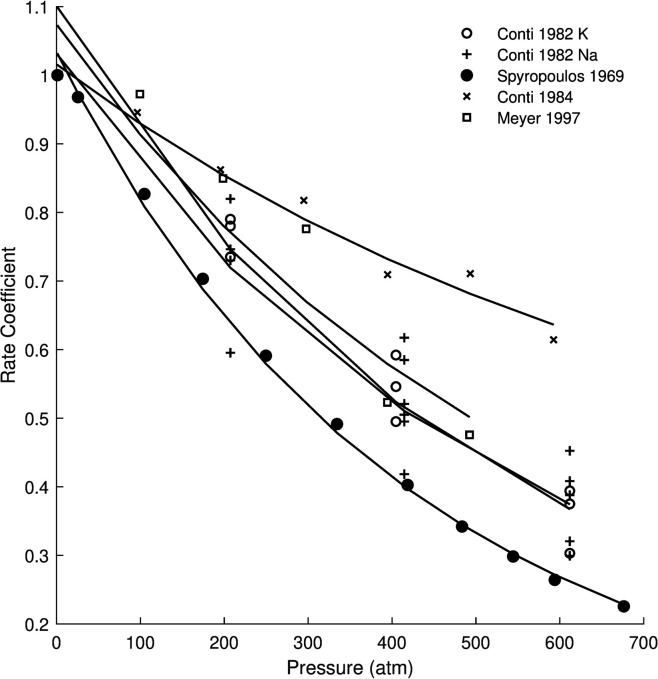
Fitting the model to experimental measurements of the rate coefficient as a function of pressure. Normalized experimental rate coefficient data [30–32,46,48,55] and model fits. See text for details.

We wanted to calculate the value of $\Delta V_{o}^{‡}$, based on electrophysiology recording and compare to our estimate. To do this we used the model to fit values of *k* extracted from experiments on different neurons and conductances [30–32,48,55], Fig 2. Depending on the source of experiments, we used the average temperature parameters we previously calculated for Na and K conductances. As we did in the past, we also fit the value of $\Delta S_{o}^{‡}$ because this varies as a function of the experimental temperature and does not affect the rate of change of the MMRT function. In all fits we assumed $\Delta {\hat{\alpha }}^{‡}=-0.20\;{\text{cm}}^{3}{\text{mol}}^{-1}{\text{K}}^{-1}$ and $\Delta {\hat{\kappa }}^{‡}=1\;\times \;10^{2}\;{\text{cm}}^{3}{\text{mol}}^{-1}{\text{K}}^{-1}$. The fits had a mean *R*^2^ of 0.93±0.06. This analysis shows very accurate values of both parameters. The average value of $\Delta S_{o}^{‡}$ was −133.97 ± 3.88 SD $\text{J}{\text{mol}}^{-1}{\text{K}}^{-1}$, with an average 95% confidence interval of 1.16±0.81 SD $\text{J}{\text{mol}}^{-1}{\text{K}}^{-1}$. The values of $\Delta S_{o}^{‡}$ were very close to those that we reported for potassium channels in our previous study. For $\Delta V_{o}^{‡}$ the average value was 40.93 ± 11.60 SD ${\text{cm}}^{3}{\text{mol}}^{-1}$ and a 95% confidence interval of 9.20 ± 6.47, which are in the range of values reported of Na and K conductances, see Table 1.

### Adiabatic heating effects on pressure measurements

**Fig 3:**
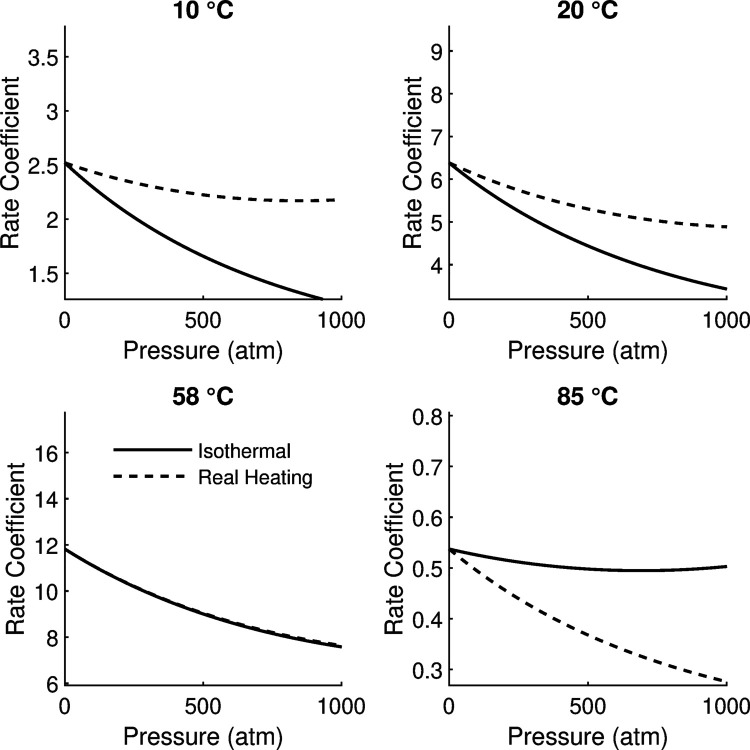
Pressure and adiabatic heating effects. The pressure dependence of rate is shown at multiple temperatures. Because increasing pressure can cause a temperature increase, we show that effect with the dashed line. The optimal temperature with the parameters used was $58^{\circ }\text{C}$ and the rates are referenced at $20^{\circ }\text{C}$.

We studied the effects of incorporating adiabatic heating in the model, Fig 3. We plotted four isotherms of the rate coefficient function using $\Delta {\hat{\alpha }}^{‡}=-0.2\;{\text{cm}}^{3}{\text{mol}}^{-1}{\text{K}}^{-1}$ and $\Delta {\hat{\kappa }}^{‡}=100\;{\text{cm}}^{3}{\text{mol}}^{-1}\text{GPa}$. We applied a one-degree increase for every 20MPa (197atm) from rapid pressure change. Depending on the starting temperature, adiabatic heating temperature change can have a significant impact on the shape of *k*. The effect of rapid heating is minimal when the pressure change is at *T*_*opt*_. At suboptimal temperatures, the heating increases rate whereas at supraoptimal temperatures heating is adverse. This behavior reflects the temperature optimum over rate and is a significant quantitative result that should be considered for rapid or transient pressure changes.

### Sensitivity to expansivity, compressibility, and activation volume

**Fig 4:**
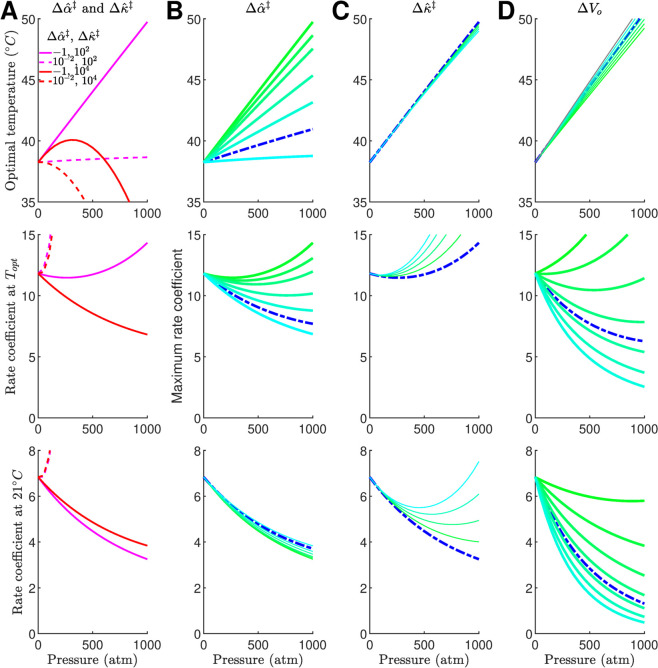
Sensitivity analysis of the rate coefficient function to pressure parameters. (**A**) The optimal temperature (*T*_*opt*_), and rate coefficient at *T*_*opt*_, and at $21^{\circ }\text{C}$ when using the combination of the extreme values of $\Delta {\hat{\alpha }}^{‡}$ and $\Delta {\hat{\kappa }}^{‡}$.(**B-D**) Sensitivity of the model to individual variations of pressure parameters. The reference model is plotted with a dash blue line in each panel. The range of values were: $\Delta {\hat{\alpha }}^{‡}$ from –1 to 0 in $0.1\;{\text{cm}}^{3}{\text{mol}}^{-1}{\text{K}}^{-1}$ increments; $\Delta {\hat{\kappa }}^{‡}$ from 100 to 500 in $100\;{\text{cm}}^{3}{\text{mol}}^{-1}{\text{GPa}}^{-1}$ increments; and $\Delta V_{o}^{‡}$ from 5 to 70 in $10\;{\text{cm}}^{3}{\text{mol}}^{-1}$ increments. The range of parameters is plotted from low to high value as green to cyan. We used as a reference the model with: $P_{o}=1\text{atm}$, $T_{o}=25^{\circ }\text{C}$, $\Delta C_{p}^{‡}=-2.76\text{kJ}{\text{mol}}^{-1}$, $\Delta S_{o}^{‡}-113\text{J}{\text{mol}}^{-1}{\text{K}}^{-1}$, $\Delta H_{o}^{‡}=34\text{kJ}{\text{mol}}^{-1}$, $\Delta V_{o}^{‡}=40.96{\text{cm}}^{3}{\text{mol}}^{-1}$, $\Delta {\hat{\alpha }}^{‡}=-0.20{\text{cm}}^{3}{\text{mol}}^{-1}{\text{K}}^{-1}$ and $\Delta {\hat{\kappa }}^{‡}=10^{2}{\text{cm}}^{3}{\text{mol}}^{-1}{\text{GPa}}^{-1}$.

In the previous sections we first calculated the value of $\Delta V_{o}^{‡}$ to avoid over-parametrization and the numerical effects on the fitting procedure of parameters with large differences in their orders of magnitude and quadratic effects of temperature and pressure. Here we perform a sensitivity analysis of the model by varying the values of $\Delta {\hat{\alpha }}^{‡}$ and $\Delta {\hat{\kappa }}^{‡}$ and $\Delta V_{o}^{‡}$, Fig 4.

We first studied how the model behaved when using the extreme values of $\Delta {\hat{\alpha }}^{‡}$ and $\Delta {\hat{\kappa }}^{‡}$, Fig 4A. We plotted the value of *T*_*opt*_, *k* at *T*_*opt*_, and at an experimental temperature, which we selected to be $21^{\circ }\text{C}$, all as a function of pressure. When using the low value of $\Delta {\hat{\kappa }}^{‡}=10^{2}\;{\text{cm}}^{3}{\text{mol}}^{-1}\text{GPa}$ we obtained a linear relationship between *T*_*opt*_ with pressure independently of the value of $\Delta {\hat{\alpha }}^{‡}$. When using $\Delta {\hat{\alpha }}^{‡}=10^{-2}\;{\text{cm}}^{3}{\text{mol}}^{-1}{\text{K}}^{-1}$ and $\Delta {\hat{\kappa }}^{‡}=10^{2}\;{\text{cm}}^{3}{\text{mol}}^{-1}\text{GPa}$, which are found in soluble proteins [39,50–54], there is a minimum effect of pressure on *T*_*opt*_. In contrast, when using the high value of $\Delta {\hat{\kappa }}^{‡}=10^{4}\;{\text{cm}}^{3}{\text{mol}}^{-1}\text{GPa}$ there is a non-linear behavior of *T*_*opt*_. The analysis of the rate coefficient at *T*_*opt*_ or at $21^{\circ }\text{C}$ suggest that there are interactions between the values $\Delta {\hat{\alpha }}^{‡}$ and $\Delta {\hat{\kappa }}^{‡}$ that result in decreasing reaction coefficient behavior as a function of pressure.

To estimate the relative effect of varying the pressure parameters we took as a reference our model parametrized with $\Delta V_{o}^{‡}=40.96{\text{cm}}^{3}{\text{mol}}^{-1}$, $\Delta {\hat{\alpha }}^{‡}=-0.20{\text{cm}}^{3}{\text{mol}}^{-1}{\text{K}}^{-1}$ and $\Delta {\hat{\kappa }}^{‡}=10^{2}{\text{cm}}^{3}{\text{mol}}^{-1}{\text{GPa}}^{-1}$, Fig 4B–4D. This shows that the behavior of *T*_*opt*_ is highly sensitive to the values of $\Delta {\hat{\alpha }}^{‡}$. The behavior of the rate coefficient is sensitive at *T*_*opt*_ but is less at our designated experimental temperature. In contrast, the behavior of *T*_*opt*_ is not sensitive to the values of $\Delta {\hat{\kappa }}^{‡}$ but could have a strong effect at experimental temperatures on the value of the rate coefficient. A similar effect is seen with the values of $\Delta V_{o}^{‡}$. Taken together, this analysis provides a methodology to distinguish between the effects of each of these parameters on how the reaction coefficient function is affected by pressure.

### Effects of temperature and pressure on action potential generation and timing

**Fig 5:**
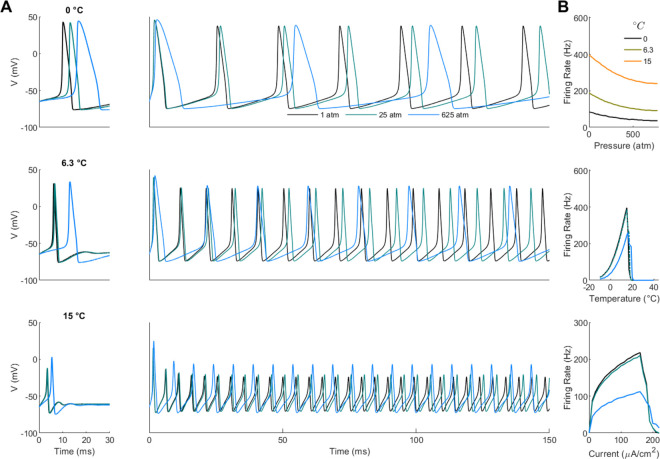
Effects of temperature and pressure on the Hodgkin-Huxley model. (**A**) left - single action potentials generated rheobase for different pressures and temperatures. Right - Spike trains under the same conditions. (**B**) Firing rate vs pressure, temperature, and input current. For each panel the pressure and temperature panels the input current was $100\mu \text{A}/{\text{cm}}^{2}$. For the bottom panel the temperature was $6.3^{\circ }\text{C}$.

We performed an analysis of the spiking and firing rate of the Hodgkin-Huxley equations under different temperature and pressure conditions, Fig 5. Increasing pressure resulted in a broadening of the action potential and a lengthening of the inter-spike interval. However, at higher temperatures, pressure had a stabilizing effect on the shape of the action potential. In all cases, we used the $\Delta {\hat{\alpha }}^{‡}=-0.2$ and $\Delta {\hat{\kappa }}^{‡}=100$, Fig 5A. The summary data, Fig 5B, shows a continuous decrease in firing rate due to pressure (top), a temperature dependence that peaks at $16^{\circ }\text{C}$, followed by a failure to generate action potentials past $21^{\circ }\text{C}$, note that we required a minimum amplitude of 20 mV to detect an action potential) (center). We also see a similar behavior of firing rate as a function of input current (bottom).

**Fig 6:**
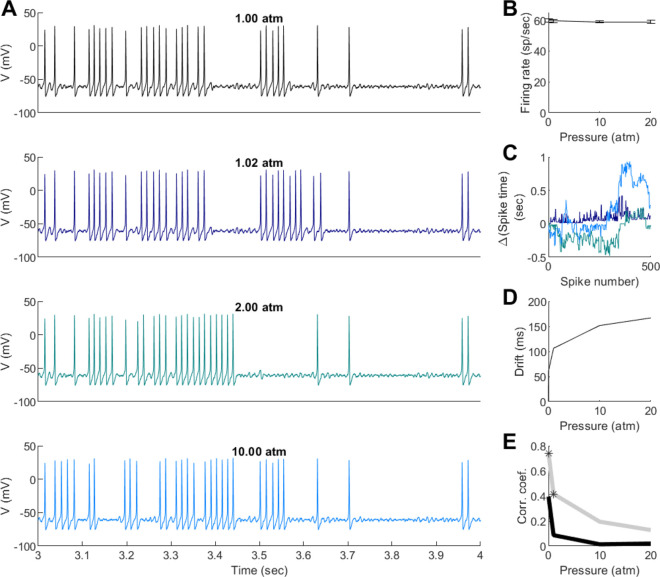
Effects of low-pressure on precise spike timing in the Hodgkin-Huxley model. (**A**) Examples of spike traces of the Hodgkin-Huxley model at different pressure receiving identical sequences of input random currents. For each value of pressure we repeated the simulations 10 times with different input current random sequences. (**B**) Average firing rate vs pressure. Error bars are for the standard deviation calculated on the 10 different runs. (**C**) Examples of spike time differences for simulations that had the same random input sequences of stimulation but different pressures (colors correspond to pressures in A). (**D**) Standard deviation of the spike time differences vs pressure. (**E**) Average correlation coefficients of the inter-spike intervals (ISI) with respect to the simulation at 1 atm (black). We recalculated the correlation coefficients after averaging 10 ISIs, showing two pressures in which the correlations were statistically significant.

The plots in Fig 5 suggest a weak pressure effect on action potential generation and average firing rate. However, in those plots, we noticed an effect on spike timing. To study the possibility that pressure could affect spike timing, but not firing rate, we performed a series of simulations in which the Hodgkin-Huxley model was stimulated with random current plus a constant component. We selected the random amplitude and DC offset to generate variable spike trains, Fig 6A. We then used the same random sequence to stimulate an identical model while varying only the pressure. We decided to study lower pressures, including one in the range of intracranial values, 0.02 atm, [35]. These simulations showed that even at very low pressures, the spiking activity could be different from the control simulation, see 1.02 vs 1.00 atm in Fig 6A. As the pressure increased, the spike trains became more different. However, the average firing rate of the entire simulation, 15*sec*, remained basically the same, Fig 6B. To evaluate changes in spiking activity, we calculated the difference in spike time from the control simulation. This pairwise calculation could be constant, corresponding to a shift in the spiking activity. However, the difference in spike time showed variability that seemed to correlate with pressure, Fig 6C. Indeed, when we calculated the standard deviation of the spike differences, there was a pressure effect. These results could be because the simulation at the higher pressure could be slowing down with respect to the control simulation with a dependence on the random noise. However, these differences remained even after averaging multiple simulations (10) using different random number sequences, Fig 6D. In order to test further the idea that the spike trains became decorrelated, we calculated the correlation coefficient of the instantaneous inter-spike interval sequences. This shows that even at the lowest pressure, the spike trains had a low and non-statistically significant correlation coefficient. We binned the ISI sequences in chunks of 10 to test if this correlation could become significant by averaging the noise. Even with this filter, there was only a significant correlation at 1.02 and 2.00 atm, Fig 6E. Together, our results suggest that small pressure changes can affect precise spike timing and correlation of spike trains across neurons.

**Fig 7:**
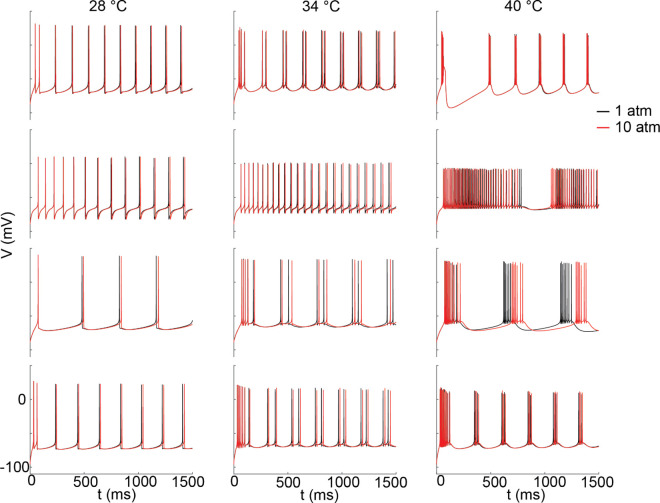
Effects of hypothermic and hyperthermic temperatures and high pressure on the spike trains of models of human cortical pyramidal cells. Rows: four different cortical models. Columns: Three different temperatures. Each model was run with normal and blast-type pressure (10 atm). The models were obtained from the Cell Types database from the Allen Institute, see text for details.

Finally, we applied the extended theory to biophysical models of human cortical pyramidal cells, see Methods. We analyzed changes in spiking over pressure ranges in blast conditions (10 atm), Fig 7. At these relatively low values of pressure changes the effects were notable in pace-making type neurons. In these and the Hodgkin-Huxley simulation we assumed steady-state temperature and pressure, and so additional temperature change from adiabatic heating was not considered. Overall, our results show that while temperature generally increases the firing rate up to *T*_*opt*_, or up to the point of over-saturation, pressure inhibits or delays action potential generation. While these effects could be mild at the single-cell level, both burst desynchronization between multiple neurons and error in very precise temporal codes could arise from pressure effects.

## Discussion

In this work, we combined concepts from MMRT and transition state theory to integrate the effects of temperature, volume, and pressure on the activation energy of voltage-gated membrane conductances. While the values of $\Delta H^{‡}$, $\Delta G^{‡}$, and $\Delta S^{‡}$ are well understood, the extended theory uses other variables and parameters that require further understanding.

### The activation volume of voltage-gated ion channels

**Fig 8:**
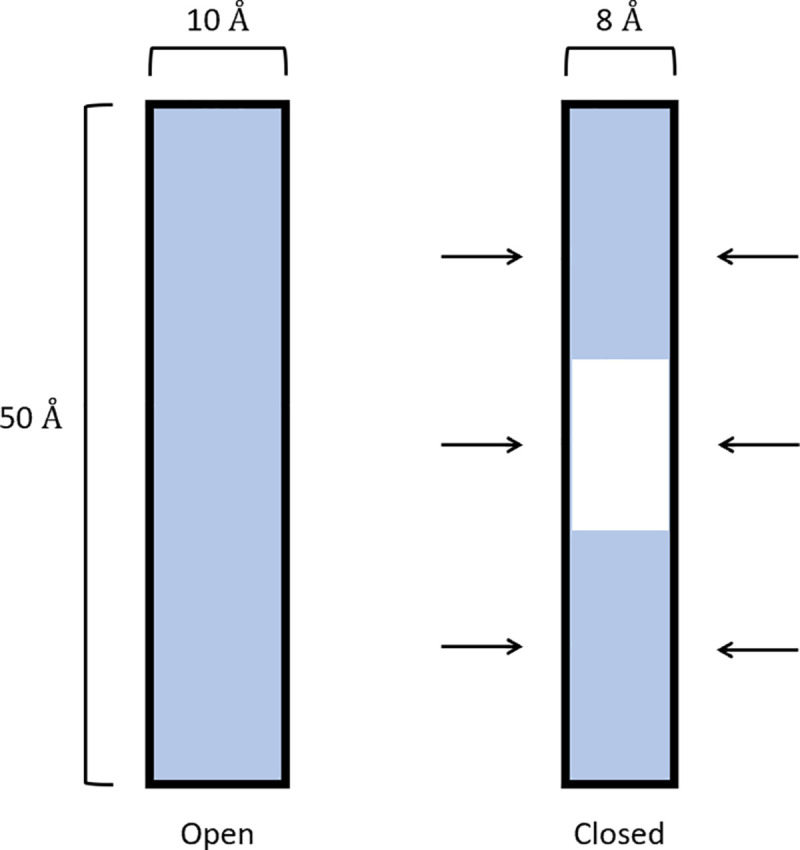
Simplified pore cross section during open and closed states. In the closed state, the radius shrinks by 1 Å which allows the fully hydrophobic part of the pore to dewet. The blue represents regions occupied by water molecules.

The length of a voltage-gated ion channel (VGIC) is around 45 Å [56–58]. The VGICs are roughly cylindrical [56,58,59] with diameters around 10 Å [56,58,60]. During opening or closing the volume of a pore may change [61–64]. The physical process of activating a channel requires dewetting that can happen by a 1-2 Å decrease in pore radius [61–63]. This simple cylindrical model is an approximation, where real channels often undergo multiple complex conformational changes which all may contribute to $\Delta V^{‡}$. Nevertheless, to gain intuition on the physical meaning of $\Delta V^{‡}$ we will assume a VGIC of height 50 Å and diameter 10 Å. We will also assume that the channel is described by a two-state process, open and closed, Fig 8. Assuming a 1 Å radial decrease when changing states, we can calculate a physical volume difference of $\Delta V^{‡}\;-\;V=2,984{Å}^{3}\approx 48{\text{cm}}^{3}/\text{mol}$ which is on the same scale as values found in multiple experiments [29–32,45–47]. This similarity between geometrically and experimentally determined values of activation volume lead us to propose that these properties could be physically modeled based on protein structure, instead of fitting and estimating them, and could be an interesting future direction of research.

### Expansivity, compressibility, and further development

Just as $\Delta V^{‡}$ allows suggestions of underlying mechanisms such as pore constriction, expansivity and compressibility may offer insights into the greater complexity behind pressure and volume effects on ion channels. For instance, compressibility is relevant to aromatic ring flip conformations of proteins [39]. Positive transition compressibility suggests that the ion channel volume is more susceptible to pressure when open. Expansivity is an important parameter as it sets the temperature dependence of activation volume. If transition expansivity is positive, one would suspect channel volume to be less partial to temperature when closed. Intuitively, this seems to contradict the concept of a closed, evacuated pore as liquid water in the open state should be less expansive. That interpretation makes negative transition values seem more plausible.

Conceptually, $\Delta {\hat{\alpha }}^{‡}$ is the difference in the partial temperature derivative of volume between the transition and ground states of a reaction. The derivatives can be large, but if we take the high value of the parameter $\Delta {\hat{\alpha }}^{‡}=-1$ and Eq 13b with our reported $\Delta V_{o}^{‡}$ we can see that just a $19^{\circ }\text{C}$ increase would set $\Delta V^{‡}=0$. Negative activation volumes are possible for processes such as unfolding [43], but are likely nonphysical for gate opening. That value also implies an extreme 100% change in activation volume. The same applies for a high compressibility of $\Delta {\hat{\kappa }}^{‡}=10,000$ which suggests activation volume would be zero after about 20atm of pressure which we know cannot be true from the experimental data. So, the *T*_*opt*_ is not a significant function of pressure.

Finally, it should be noted that $\Delta C_{p}^{‡}$, $\Delta {\hat{\alpha }}^{‡}$, and $\Delta {\hat{\kappa }}^{‡}$ may all depend on the pressure and temperature themselves. We mentioned that $\Delta C_{p}^{‡}$ is assumed constant, but it could be given a linear temperature dependence [17], some parameter-based function of temperature and pressure [65], and it has been suggested to increase with pressure [66]. If $\Delta C_{p}^{‡}$ had a significant pressure dependence, it could also explain the apparent temperature dependence of $\Delta V^{‡}$, rather than or in addition to $\Delta {\hat{\alpha }}^{‡}$. For simplicity, we did not include adiabatic heating in our simulations. Any of these effects, if significant at a biological temperature range or at high pressures, would limit the model at its current level of detail, but not necessarily invalidate the modeling strategy. Though, outside of biological ranges, where there is protein denaturation or cell death, the model regardless of parameters would certainly no longer apply.

## Conclusion

We presented thermodynamic theory that can be integrated with studies of neuronal excitability [67,68]. More broadly, a unified temperature and pressure theory can be used to compare the enzymatic kinetics of diving creatures and extremophilic bacteria in hundreds of atmospheres in the ocean [20,21,34] with land creatures or their ancestors. Our work provides a platform to study the evolution of preferred body temperatures [33] with the optimal temperature of enzymes. Pressure has mechanical consequences for neuronal function and structure [69,70] due to membrane mechanics. In the context of human health, changes in intracranial pressure (ICP) can arise from many phenomena such as intracerebral hemorrhage [71], plateau waves [72], microgravity [73], and impacts and blasts [35,36]. For example, acceleration effects can change the ICP and have been shown to have negative effects on cognitive performance [74,75]. Pressure changes caused by ICP are around 10-100 mmHg (0.05atm) which we showed have little effect on the firing rate [76], but that could affect precise spike timing. It remains to evaluate in a similar way if the small pressure changes from action potential propagation [9] could have a significant effect. In any case, our work suggests that pressure affects precise spike timing and we suggest that cumulative effects could modify network dynamics and performance. The mechanisms we describe could combine with other cellular communication mechanisms important for network activity, such as the effects of pressure on synaptic release [47]. Other areas to consider are how membrane thermodynamics affect anesthesia [37,38] and how membrane changes could alter channel function [7]. While in this study we focused on developing theory for applications on neuronal intrinsic excitability driven by voltage activated ion channels there could be other emergent network-level effects or intracellular metabolic pathways that together could enrich the thermodynamical effects on neuronal and network function. As such, our work promotes the return to experimentation and discussion of pressure, especially since pressure experiments can reveal important biological properties [77].
